# Using NT-proBNP to Detect Chronic Heart Failure in Elderly Patients with Chronic Obstructive Pulmonary Disease

**DOI:** 10.5402/2013/273864

**Published:** 2013-06-09

**Authors:** Elzbieta Kaszuba, Bartlomiej Wagner, Håkan Odeberg, Anders Halling

**Affiliations:** ^1^Blekinge Competence Centre, Wämö Centre, 371 81 Karlskrona, Sweden; ^2^Lund University, Department of Clinical Sciences in Malmö, General Practice/Family Medicine, 205 02 Malmö, Sweden; ^3^Research Unit of General Practice, Institute of Public Health, University of Southern Denmark, 5000 Odense C, Denmark

## Abstract

*Objective*. To detect chronic heart failure in elderly patients with a registered diagnosis of chronic obstructive pulmonary disease (COPD) treated in Swedish primary health care using natriuretic peptide NT-proBNP. *Design*. A cross-sectional study. *Setting*. Two primary health care centres in southeastern Sweden each with about 9000 listed patients. *Subjects*. Patients aged 65 years and older with a registered diagnosis of COPD. *Main Outcome Measures*. Percentage of patients with elevated NT-proBNP, percentage of patients with abnormal left ventricular function assessed by echocardiography, and association between elevated NT-proBNP and symptoms, signs, and electrocardiography. *Results*. Using NT-proBNP threshold of 1200 pg/mL, we could detect and confirm chronic heart failure in 5.6% of the study population with concurrent COPD. An elevated level of NT-proBNP was only associated with nocturia and abnormal electrocardiography. *Conclusions*. We found considerably fewer cases of heart failure in patients with COPD than could be expected from the results of previous studies. Our study shows the need for developing improved strategies to enhance the validity of a suspected heart failure diagnosis in patients with COPD.

## 1. Introduction 

When a general practitioner encounters an elderly patient with chronic obstructive pulmonary disease (COPD) presenting with shortness of breath, cough, and greater fatigue than usual, it is easy to suspect COPD exacerbation. However, these symptoms are unspecific and may also be consistent with a heart failure diagnosis. 

Prevalence of chronic heart failure in elderly patients with COPD carried in a primary health care was found to be 20.5% [1]. A review of previous studies showed that prevalence of heart failure or left ventricular systolic dysfunction in patients with COPD varied between 10% and 46% [2]. The highest prevalence of chronic heart failure was reported among patients with symptomatic dyspnoea [3]. 

Most patients with COPD and/or chronic heart failure in Sweden are managed in primary health care. Assessment of probability of chronic heart failure by judgment of clinical symptoms in the same patients differs considerably among general practitioners [4]. Similarities in signs and symptoms make it more difficult to diagnose chronic heart failure in patients with COPD. Access to echocardiography in primary health care in Sweden is limited; therefore, this essential diagnostic tool is not immediately available in routine care. Determination of natriuretic peptides constitutes an important component of the European Society of Cardiology (ESC) algorithm for the diagnosis of chronic heart failure [5].

Natriuretic peptides enhance validity of heart failure diagnosis in elderly patients with COPD [6]. The aim of the present study was to evaluate if the analysis of NT-proBNP might be used as an initial step for the diagnosis of chronic heart failure in patients with COPD in primary health care and to select patients for a further examination by echocardiography.

Furthermore, the patients with elevated NT-proBNP were compared with the other participating patients for different symptoms and electrocardiographic abnormalities. 

## 2. Material and Methods

The study was conducted in two different primary health care centres during the period 16 April 2008–13 June 2008 in Blekinge county. Blekinge county with approximately 150 000 inhabitants is located in southeastern Sweden. Olofström municipality has 13 198 inhabitants, with 22.7% 65 years and older. Karlskrona municipality has 62 338 inhabitants with 19% 65 years and older [7]. 

The study included patients aged 65 years and older with the following diagnosis codes according to ICD 10: J44 (COPD), J41 and J42 (chronic bronchitis) registered during the period 1 January 2008–16 April 2008 according to the electronic patient record.

**Figure 1:**
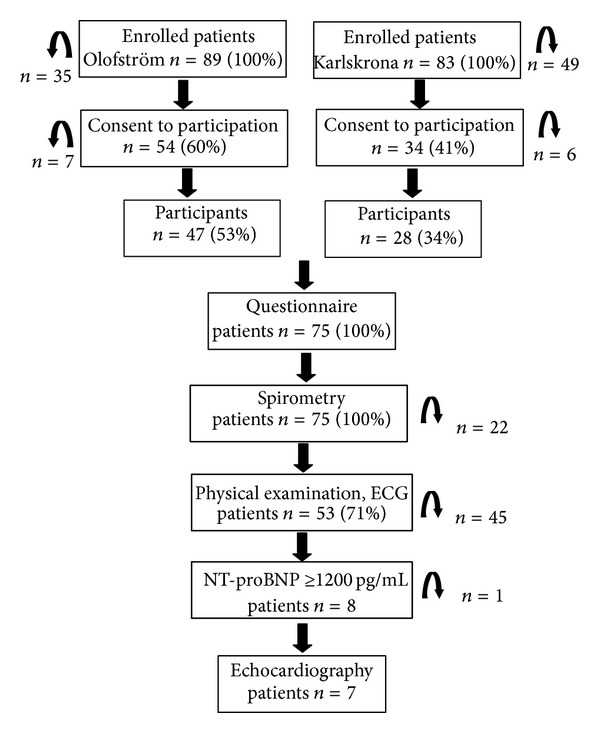


Data on the patient flow during the study are shown in Figure 1. 

At the time of the study, 9265 patients were registered at the primary health centre in Olofström, 1905 (20.6%) aged 65 years and older. At the primary health centre Tullgården in Karlskrona, 9002 patients were registered and 1508 (16.8%) aged 65 and older. 

Exclusion criteria comprised impaired cognitive function and/or anticipated difficulties in carrying out spirometry due to immobility, psychiatric disorders, or terminal illness.

 Informed consent was obtained from each participant. 

In order to confirm the diagnosis of COPD and classify the degree of severity of COPD according to the GOLD criteria [8], all participants were examined with dynamic spirometry (Spirare 3, Diagnostica, Norway), if spirometry had not already been carried out during the past year. 

Patients with a confirmed diagnosis of COPD were examined regarding chronic heart failure. 

 In an interview, we asked the patients for the following symptoms: breathlessness, orthopnoea, night cough, nocturia, and walking distance. A threshold for walking distance of 100 meters was used [9]. Physical examination included weight and height, heart and lung auscultation, blood pressure measurement after 5 minutes' rest in the sitting position, and the presence of peripheral oedema. Positive findings from heart and lung auscultation were determined as rales and the third heart sound.

 All patients were examined by electrocardiography. 

Since the regional ethics committee questioned the need to perform chest X-rays in all participants because of the risk associated with radiation, a chest X-ray was performed only if clinical examination gave rise to suspicion of pulmonary congestion. 

The natriuretic peptide was determined as NT-proBNP (Immulite 2500, Siemens Healthcare Diagnostics AB, Sweden). Patients with the NT-proBNP level of ≥1200 pg/mL were referred for echocardiography to assess left ventricular (LV) function using the following echocardiographic criteria:

- EF ≥55% normal systolic LV function,
- EF 40–54% mildly impaired systolic LV function,
- EF 30–39% moderately impaired LV function,
- EF <30% severely impaired LV function.

Abnormal LV relaxation and/or distensibility during normal EF were described as diastolic dysfunction. 

The study was approved by the research ethics committee at Lund University.

The study was registered at ClinicalTrials.gov NCT01801722.

## 3. Statistics

Data were analysed in the STATA version 10 (Stata Corporation, Texas, USA). Distribution of categorical variables is presented as numbers. Distribution of continuous variables was presented as mean and standard deviations (SD) except NT-proBNP which was presented as the median and interquartile range (IQR). Mann-Whitney's test was used for comparison of mean values. Differences in proportions in the groups were tested with the chi square test. A *P* value of <0.05 was considered significant.

## 4. Results

The mean age of the 75 participating patients was 75.3 (SD 7.6), while the age of 108 patients who did not participate was 77.2 (SD 7.7). There was no significant difference in age (*P* = 0.09) or gender (*P* = 0.16) distribution between those groups. 

Dynamic spirometry in the participating patients showed FEV 1 >65% in 22 of the 75 patients (29%). As the diagnosis of COPD could not be confirmed in these 22 patients, they were excluded from the study. 

Statistical analysis was done on the 53 participating patients with confirmed COPD. 

The group comprised 25 women (47%) and 28 men (53%). The mean age was 75.4 years (SD 7.9), 76.3 (SD 7.6) for men, and 74.4 (SD 8.2) for women.

Spirometry showed COPD in stage 1 in 10 patients (19%), stage 2 in 28 patients (54%), stage 3 in 11 patients (21%), and stage 4 in 1 patient (2%). The severity of COPD could not be classified in 2 patients (4%) as they were older than 90 years and no reference of FEV1 is available for this age group. A median of NT-proBNP was 228 pg/mL (IQR 89–561). 

**Figure 2:**
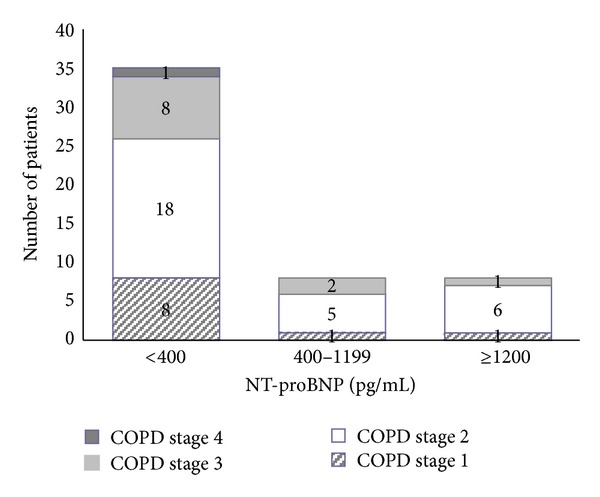
Distribution of NT-proBNP in relation to COPD stage.

Distribution of NT-proBNP in relation to COPD stages is presented in Figure 2. 

An NT-proBNP level equal to or above 1200 pg/mL was found in 8 out of 53 patients (15%).

These 8 patients were significantly older than the other participants (*P* = 0.03). We did not find a correlation between the NT-proBNP level and COPD stage (*P* = 0.9). 

Patients with an increased level of NT-proBNP (≥1200 pg/mL) were classified as having suspected chronic heart failure and referred for echocardiography. One person died while awaiting examination. Only 2 out of 7 patients (28%) with suspected chronic heart failure had a reduced EF (20 and 35%). One out of 7 patients (14%) had signs of diastolic dysfunction. We were only able to confirm the diagnosis of chronic heart failure in 3 out of 53 patients, which constitutes 5.6% of the study population. 

Correlations between symptoms, clinical findings, and NT-proBNP are presented in Table 1. Nocturia was the only registered symptom found more commonly among patients with suspected heart failure, that is, with NT-proBNP ≥1200 pg/mL. We found a strong correlation between elevated NT-proBNP level and an abnormal electrocardiography (*P* = 0.007). 

The abnormalities found were the following: complete or incomplete left bundle branch block, pathological Q wave, ST-T changes, atrial fibrillation, and signs of left ventricular hypertrophy. 

## 5. Discussion 

The purpose of this study was to evaluate whether the determination of NT-proBNP can help a general practitioner to make a valid diagnosis of chronic heart failure in patients with COPD. Using the threshold of NT-proBNP of ≥1200 pg/mL, we found that 5.6% of the participating patients had chronic heart failure. We found considerably fewer cases of chronic heart failure than could be expected from the results of previous studies. 

We chose elderly patients with COPD as a study population due to the previously reported higher prevalence of chronic heart failure in this group [10]. 

Data are also available showing that the risk of developing chronic heart failure in elderly patients with COPD is at least two times higher than in age-matched controls [11]. 

We do not believe that the low prevalence of chronic heart failure found in our study is due to a generally low prevalence of chronic heart failure in the Swedish population. Sweden has the highest proportion of elderly in the world and prevalence of chronic heart failure in Sweden is comparable with other countries [12]. 

The limitation of our study is a high drop-out rate. Firstly, the response ratio was low and the participation ratio differed considerably between participating primary health centres. 

We hypothesized that this could depend on the specialist care at the Blekinge County Hospital. This statement particularly concerned primary health centre in Karlskrona due to the vicinity of the hospital. However, when we explored hospital diagnosis registers in 2008, we found only 15 patients from primary health centre in Karlskrona and 11 patients from primary health centre in Olofström with the diagnosis of COPD. Moreover, we only found 2 patients with a combined diagnosis of COPD and chronic heart failure, both from Karlskrona.

Secondly, definite COPD could not be diagnosed in 22 (29%) of participants. The number of patients with an unconfirmed COPD diagnosis was 37.8% in a primary health centre study in The Netherlands [3].

The main question in our study was if testing of NT-proBNP can help general practitioner select COPD patients for a confirmatory investigation of chronic heart failure using echocardiography. Symptoms and signs were of no help in the diagnosis of chronic heart failure, except probably for nocturia, which was more frequent in COPD patients with elevated NT-proBNP. There was a strong correlation between an abnormal ECG and an elevated NT-proBNP, which confirms the fact that a normal ECG makes diagnosis of chronic heart failure less likely [13]. 

An elevated level of natriuretic peptides is an important component of the ESC diagnostic algorithm. Two threshold values are used. The diagnosis of chronic heart failure is considered unlikely below 400 pg/mL, likely above 2000 pg/mL, and uncertain in between. The level of natriuretic peptides can be increased in patients with COPD [14]. Age is one of the main factors influencing the NT-proBNP level [15]. We chose an NT-proBNP level of 1200 pg/ml as a reasonable level at which to suspect chronic heart failure in elderly patients with COPD.

 The value of 1200 pg/mL also constitutes the mean of the two threshold levels of the ESC algorithm. If the lower threshold level of 400 pg/mL had been used in our study, 17 out of 53 patients (32%) would have been eligible for echocardiography. Only 3 out of 7 (42%) patients with levels ≥1200 pg/mL had abnormal findings in echocardiography. Furthermore, their NT-proBNP levels were rather high: 1682 pg/mL, 1877 pg/mL, and 4413 pg/mL, indicating that the small number of patients found with abnormalities in echocardiography was not explained by a too high threshold of NT-proBNP.

The natriuretic peptides can be used in the general population as a tool to evaluate left ventricular systolic dysfunction [16] and to make a diagnosis of heart failure more accurate [17, 18]. A newer study from Denmark suggests that NT-proBNP can be used to risk stratify waiting lists for echocardiography in PHC [19]. There is a possibility that NT-proBNP used for screening patients eligible for echocardiography is not suitable when patients with suspected chronic heart failure also have COPD. Improving strategies to enhance validity of the chronic heart failure diagnosis in patients with COPD should be of interest for general practitioners. Our study is small and a more extended study is needed to settle this issue from the primary health care perspective. 

## Figures and Tables

**Table 1:** Correlations between symptoms, clinical findings, and NT-proBNP.

	NT-proBNP	<1200 *n* = 45	≥1200 *n* = 8		*P* value
	Positive	Negative	Positive	Negative	
Symptoms					
Breathlessness	37 (82%)	8 (18%)	7 (87%)	1 (13%)	0.714
Night cough	8 (18%)	34 (76%)	0 (0%)	8 (100%)	0.178
Orthopnoea	3 (7%)	39 (86%)	1 (13%)	7 (87%)	0.609
Nocturia	31 (69%)	14 (31%)	8 (100%)	0 (0%)	0.066
Walking distance	13 (29%)	26 (58%)	1 (13%)	6 (85%)	0.313
Clinical findings					
Heart auscultation	3 (7%)	42 (93%)	1 (13%)	7 (87%)	0.565
Peripheral oedema	21 (47%)	24 (53%)	3 (37%)	5 (63%)	0.631
ECG abnormalities	22 (49%)	23 (51%)	8 (100%)	0 (0%)	0.007
